# To be seen, confirmed and involved - a ten year follow-up of perceived health and cardiovascular risk factors in a Swedish community intervention programme

**DOI:** 10.1186/1471-2458-7-190

**Published:** 2007-07-31

**Authors:** Maria Emmelin, Lars Weinehall, Hans Stenlund, Stig Wall, Lars Dahlgren

**Affiliations:** 1 Epidemiology and Public Health Sciences, Department of Public Health and Clinical Medicine, Umeå University, Umeå, Sweden; 2 Centre for Population Studies: Ageing and Living Conditions Programme, Umeå University, Umeå, Sweden; 3 Department of Sociology, Umeå University, Umeå, Sweden

## Abstract

**Background:**

Public health interventions are directed towards social systems and it is difficult to foresee all consequences. While targeted outcomes may be positively influenced, interventions may at worst be counterproductive. To include self-reported health in an evaluation is one way of addressing possible side-effects. This study is based on a 10 year follow-up of a cardiovascular community intervention programme in northern Sweden.

**Methods:**

Both quantitative and qualitative approaches were used to address the interaction between changes in self-rated health and risk factor load. Qualitative interviews contributed to an analysis of how the outcome was influenced by health related norms and attitudes.

**Results:**

Most people maintained a low risk factor load and a positive perception of health. However, more people improved than deteriorated their situation regarding both perceived health and risk factor load. "Ideal types" of attitude sets towards the programme, generated from the interviews, helped to interpret an observed polarisation for men and the lower educated.

**Conclusion:**

Our observation of a socially and gender differentiated intervention effect suggests a need to test new intervention strategies. Future community interventions may benefit from targeting more directly those who in combination with high risk factor load perceive their health as bad and to make all participants feel seen, confirmed and involved.

## Background

Public health interventions are directed towards social systems trying to influence people's attitudes and actual behaviours. They aim to create a positive infrastructure for change and a will to initiate action on both community and individual level [1]. However, social systems are vulnerable and it is difficult to foresee all consequences of an intervention. While targeted outcomes may be positively influenced, intervention strategies may also have unintended consequences and at worst be counterproductive for example by increasing the risk of stigmatisation, labelling or discrimination [2]. The effects can also differ unfairly by age, gender and educational level. To include self-reported outcome measures of health is one way of addressing these possible side-effects.

Through a few simple questions self-rated health indicate how respondents perceive their health in general and/or in comparison with other people of their own age. Self-rated health is known to be a multi-dimensional concept including not only physical aspects of health but also functional, coping and well-being (mental/emotional) dimensions [3]. It is an inexpensive instrument and the reliability has been shown to be high in all social strata [4,5]. A review of a total of 46 community studies concluded that self-rated health is an independent predictor for survival even when other health status indicators are taken into account [6,7]. However, there is still a debate about the relative importance of underlying medical and social variables in predicting future ill-health or mortality. There is also a need of a deeper understanding about how these variables are related to perceptions of health in different social or cultural settings [8-14].

Few studies have focused on self-rated health as an outcome of public health interventions. The North Karelia study has shown that the self-rated good health ratings improved significantly more in the intervention than in the reference area and that the perceived risk of developing cardiovascular disease decreased [15]. However, we found no studies specifically focussing on how self-rated health combines with risk factor changes as outcome measures for cardiovascular disease intervention programmes. Also, self-rated good health may contribute to a better prognosis of future risk factor burden.

In 1985 a community intervention programme was launched in the municipality of Norsjö in northern Sweden. This was a demonstration and feasibility project for measuring effects of cardiovascular disease prevention strategies before disseminating a programme to the whole county. The Norsjö Municipality Board as well as community members had expressed their concern and turned to the County Council with a request for public health initiatives because cardiovascular diseases had been identified as a major public health problem in the area [16]. The project was designed to combine a population strategy with efforts to meet, examine and give health advice individually to people when they were 30, 40, 50 and 60 years of age. Using the primary care system as a partner in the community intervention, the programme carried out systematic risk factor screening and individual counselling by its family medicine providers while involving the whole municipality in different strategies to raise public awareness.

The evaluation of the Norsjö programme has included different disciplines and methodologies. Risk-factor changes have been studied in relation to the community participation process [17], health economic analyses have focussed on the equity aspects of the program [18] and the role of the primary health care system in the risk factor reduction has been assessed [19]. The observed positive cardiovascular risk factor reduction and the results from the process evaluation have been guiding the intervention strategies in the county and in 1991 the programme was implemented in varying forms in the whole region as the Västerbotten Intervention Programme (VIP). The programme has gained international interest for comparisons of cardiovascular prevention strategies [20] and for its attempt of dissecting the "black box" of community interventions in general [1].

Self-rated health was included in the survey questionnaire from the start. During the first six years of intervention, based on cross-sectional surveys, people in the study area were shown to have a less favourable perception of their health than those in the reference area. However, the difference did not remain after accounting for sex, age and emotional and social support [21]. A significant association was observed between increased cardiovascular risk factor burden and self-rated health for both men and women [22]. Women, according to their own evaluation, had changed health behaviour more than men and young men perceiving ill-health had been hardest to reach [23]. Case-referent studies from the study area, but not directly related to the evaluation, analysed the interaction between self-rated poor health and bio-medical risk factors in predicting future disease. Self-rated ill-health increased the risk of future acute myocardial infarction (AMI) fivefold for those with a high risk factor burden [24]. For stroke, self-rated ill-health strengthened the effect of bio-medical risk factors, especially for men [25].

A need to further analyse how perceived health interacts with the risk factor outcome of a community intervention programme was identified. The individual changes of risk factor load and people's self-rated health and their interaction have therefore been studied in a longitudinal panel approach. To be able to discuss the mechanisms through which perceived health and risk factors are influenced by the intervention process we also needed to know more about the health related norm system in the study area. Therefore people's views and perceptions of health, illness and risk factors as well as their attitudes towards the community intervention as such were addressed.

The specific aims of this study are:

- to describe changes in self-rated health during a 10 year intervention period and analyse how these changes are related to changes in risk factor load, accounting for possible gender and educational differences

- to describe health related norms and attitudes embedded in the social context and discuss their influence on attitudes and feelings towards the intervention programme and the observed development of self-rated health and risk factor load

The study was approved by the Research Ethics Committee at Umeå University and the data handling procedures by the National Computer Data Inspection Board.

## Methods

### Triangulation

**Figure 1:**
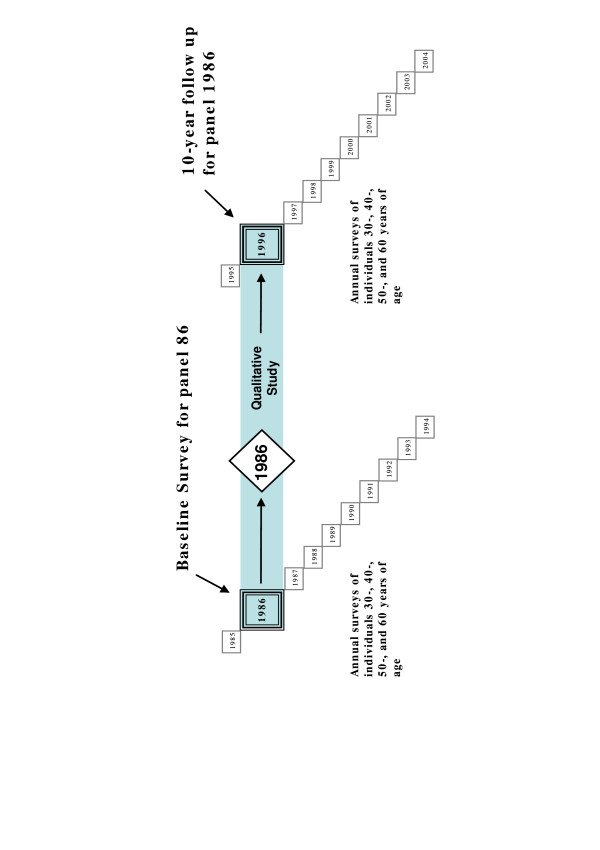
The context of the 1996 panel study within the Västerbotten Intervention Programme (VIP).

To address individual changes in risk factor load and self-rated health in relation to health related norms and attitudes we combined quantitative and qualitative approaches. Survey data for those who had been exposed both to the community intervention activities and the health provider risk factor screening and counselling in 1986 and 1996 were supplemented with additional questionnaire data on health perceptions and with qualitative research interviews (Figure 1).

### The surveys

As an integral part of the community based activities all persons in Norsjö, at the age of 30, 40, 50 and 60 were invited to a health examination at the primary health centre focussing on the traditional risk factors for cardiovascular disease. This was also an opportunity for health communication where all individuals were given verbal information and counselling about their test results and counselling and they were encouraged to participate in the community based activities. In connection with the health examination participants were also asked to complete a questionnaire with socio-demographic as well as health behaviour related questions. These cross-sectional surveys have been ongoing activities in Norsjö since 1985. As everybody is invited every ten years data on individual 10 year follow-up are available from 1995. For this study we decided to form a panel of participants from the 1986 health survey. When they were re-invited in 1996, the follow-up survey was therefore expanded to include also those turning 70 years. The questionnaire was supplemented with questions related to subjective well-being, attitudes and perceptions related to health and illness.

The total number of participants in the 1986 cross-sectional survey was 260 out of 272 invited (96%). In 1996, 11 persons of the 260 had moved from Norsjö, 17 had died, 46 did not show up and 12 could not be matched because they had only participated in the measurement part in 1986. Thus a total of 174 participants, 67% of the initial 260, had taken full part in both surveys and forms the panel group for this study. The participants were somewhat older than the non-participants, i.e. 53% belonged to the older age group compared to 47% of the non-participants. Among the younger non-participants males were overrepresented (55% compared to 45% among participants). For education as well as for the included biomedical risk factors there were no significant differences between participants and non-participants. Due to missing data some analyses are based on fewer than the 174.

### The qualitative research interviews

To capture health related norms and attitudes we made a purposive sampling of key informants. The informants were expected to inform us not only about their own health related attitudes and experiences of the programme but also about the changes of community and collective norms and values over time. Thus, during late 1995 and early 1996, we asked the primary care unit in Norsjö to help us approach participants that were soon to be invited for a 10 year follow-up. The prerequisite was that they should have participated in the health examination in 1986, that they represented some variation in age and sex, were able to reflect back and were expected to have had different experiences from the health examinations. The first two interviews formed the basis for the continued sampling of informants to capture the range and variation of experiences. In total nine interviews were performed with two men and seven women. The men were 60 years and of the women one was 60, two were 50 years and two were 40 years during the first health examination. The interviewed men brought their wives as they saw their experiences of the program as a joint venture. The interviews took between 1.5 to 2 hours and were performed by one or two members of the research group. Due to the informants' preference, interviews took place at the primary health centre. The interview guide included themes to be covered, such as views on health, health related norms and attitudes, experiences (feelings, reactions, behavioural change) during the intervention and reflections about its impact both on individual and community level over time.

### Informed consent

Participants in the surveys and interviews were informed individually about the study objectives and gave their informed consent prior to participation.

### Classification of variables

#### Biomedical risk factors

*Body Mass Index *(BMI) was calculated as weight (kg)/height(m)^2^. When dichotomised for calculation of risk factor load, high BMI (obesity) was defined as ≥ 30.

*Hypertension *was defined as systolic blood pressure ≥ 160 mmHg and/or diastolic blood pressure ≥ 95 mmHg (according to WHO guidelines at the time of the study) or reported use of antihypertensive medication during a period of 14 days before the health survey.

*Smokers *were defined as those reporting daily smoking of cigarettes, cigarillos, cigars or pipe. Ex- smokers or "occasional smokers" were classified as non-smokers.

*Hypercholesterolaemia *was defined as total serum cholesterol ≥ 6.5 mmol/l.

*Risk factor load *was calculated by adding the presence of any of the risk factors (smoking, hypertension, cholesterol ≥ 6.5 and a Body Mass Index (BMI) ≥ 30), into a score of zero to four. The load was dichotomised into low or high risk factor load, where low load was defined as having 0 or 1 of the included risk factors and high load as having 2 or more risk factors.

#### Socio-demographic factors and self-rated health

The socio-demographic factors included in the analysis are sex, age group and educational level.

*Educational level *was defined as total years at school and was dichotomised into low (up to 9 years) and high education (10 years and above).

*Self-rated health *(a proxy for perceived health) was based on a survey question about how the respondents graded their general health. In 1986 they were given three and in 1996 five response alternatives to choose from, ranging from good to bad. In the analysis a dichotomized variable was used. "Good" corresponds to the response alternatives very good and pretty good for the 5 grade and good for the 3 grade scale. "Bad" refers to fair, fairly poor and poor for the 5 grade and fair and poor for the 3 grade scale.

#### 1996 survey questions on subjective well-being and health related attitudes

In this paper we have focussed on three sets of questions where the respondents were asked to a) rank the importance (1–5) of pre-determined aspects of health (see Table 2), b) state to what extent, from yes, most probably(1) to not at all (5), they think it is shameful to be long-term sick and c) locate themselves in a two by two table on the basis of being sick or not and of feeling good or bad.

**Table 2:** Participants in the 1996 health survey rating certain aspects as important for their health

**Aspects of health**	**Percent rating this aspect as very important for health (1 or 2 on a scale 1–5)**					
	
	Men	Women	High education	Low education	40–50 years	60–70 years
Never being ill or having a disease	49	61	60	57	58	53
Being physically fit and having energy	54	49	50	55	60	44
Being psychologically fit	35	27	45	20	36	27
Being capable of performing daily tasks	24	24	20	28	22	26
Leading a healthy life style	14	14	13	15	12	16

### Analysis

#### Statistical methods

We performed multivariate logistic regression analysis using a combination of self-rated good health and low risk factor load in 1996 as the outcome variable. The main background variable in the regression was a combination of self-rated health and risk factor load in 1986. The variables adjusted for were sex, educational level and age group.

Internally missing data for the risk factors, cholesterol (2/84 for men and 3/90 for women), blood pressure (0/84 for men and 2/90 for women) and BMI (2/84 for men and 0/90 for women) were replaced with mean values. Missing value for smoking was categorised as non smoking. For educational level, missing data for non-skilled workers were classified as low educational level. Remaining missing data were included as a separate category in the model but not reported in the presentation. For self-rated health missing data were regarded as good health.

#### Qualitative analysis

All interviews were transcribed, coded and sorted using the OpenCode software [26]. The interpretation followed the basic steps of Grounded Theory [27], where the open coding resulted in a decision to focus on certain concepts capturing norm systems and attitudes to the intervention programme. The interviews were re-read, summarised, compared and re-coded in a selective coding process. The presentation of the health related norm systems is mainly descriptive using quotes from the interviews to illustrate the observed patterns. In the analysis of the attitudes towards the intervention programme we initially generated codes about feelings towards the programme. These codes are often characterised as "in vivo", meaning that they are suggested directly from what the informants express. In the next step we coded for more cognitive components of the attitudes towards the programme. These codes together guided us in a search for more abstract categories of theoretical relevance. The analysis included categorisation of these attitude sets into a typology of how the intervention programme may have interacted with health related norm systems and thus influenced both perceptions of health and risk behaviour. We constructed what the sociologist Weber has labelled, ideal types [28]. These are theoretical constructs that in the shape of metaphors aim at capturing what the attitudinal set represent. In contrast to Weber's logically constructed types ours are grounded in empirical data. This implies that one informant can contribute data to several of the ideal types.

## Results and discussion

### Ten year follow-up of risk factor load and self-rated health

Table 1 shows the distribution of socio-demographic characteristics as well as cardio-vascular risk factors and self-rated health in 1986 and 1996 among the panel participants (n = 174) that that constitute the basis for this analysis.

**Table 1:** Frequency distribution of characteristics for panel participants (n = 174)

Characteristics	1986	1996
Gender		
Men	84	84
Women	90	90
Age group		
30–40	82	82
50–60	92	92
Education		
≥ 10 years	79	-
<9 years	78	-
Missing	17	-
BMI		
BMI < 30	153	137
BMI ≥ 30	21	37
Hypertension		
No Hypertension	129	126
Hypertension	45	48
Smoking		
Non smokers	144	156
Daily smokers	30	18
Cholesterol		
No Hypercholesterolaemia	80	132
Hypercholesterolaemia	94	42
Risk factor load		
Low risk	118	135
High risk	56	39
Self-rated health		
Good	119	126
Bad	55	48

#### Changes in risk factor load and self-rated health

Other studies have evaluated the risk factor reduction in the Norsjö intervention area compared to a reference area up to 1992/94 [29,30]. Our present study confirmed a risk factor load reduction between 1986 and 1996. The risk factor load improved from 68 % having a low load in 1986 compared to 78% in 1996. Only 12% of those with low load in 1986 moved to the high risk load group while 55% moved from high risk factor load to low. All risk factors were involved in the risk reduction. However, the greatest impact was seen for cholesterol where as many as 63% with a high level at baseline had low levels at follow-up. Smoking decreased by 60%, hypertension with 38% while only 14% reduced their BMI. Studies on the general trends in cardio-vascular risk factors from the Northern MONICA study (reference area for the intervention) during the period 1986–1999 support a positive intervention effect, especially regarding cholesterol. Cross sectional survey data indicated that the proportion with high levels of cholesterol decreased from 41% in 1986 to 26% in 1999 [31]. In our intervention panel the proportion decreased from 54% at baseline to 24 % at follow-up; a much greater change, taking also into account that the panel is growing older. The proportion of daily smokers also decreased in the general area but not as much as in the intervention area [32].

**Figure 2:**
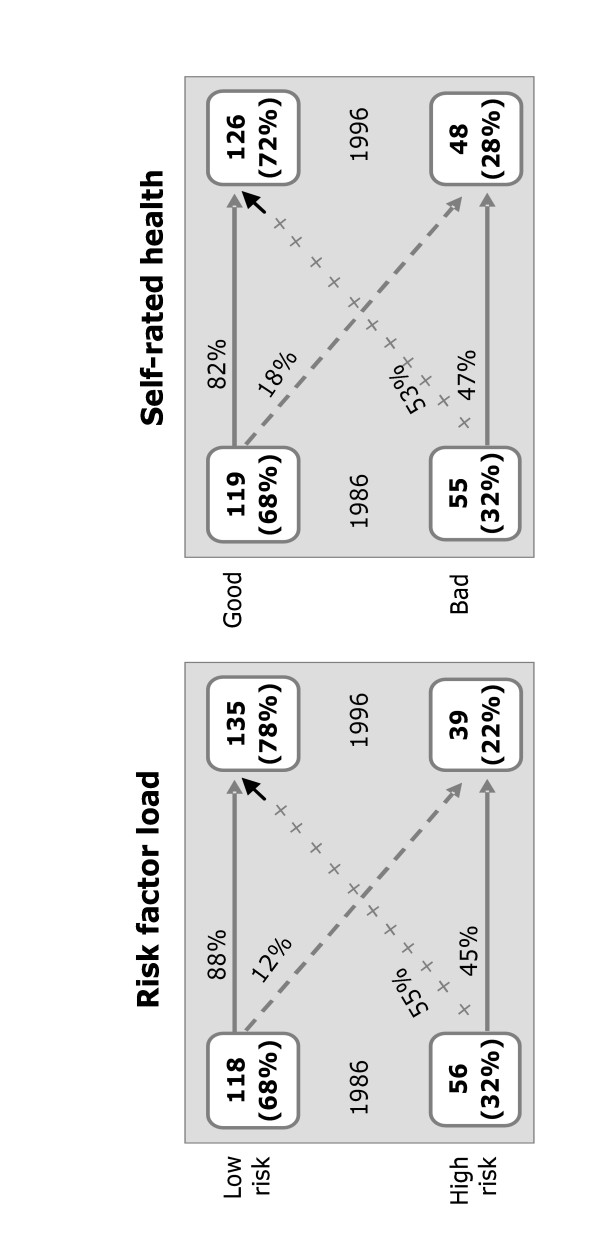
The distribution of risk factor load and self-rated health in 1986 and 1996 and the corresponding movements between the groups among the panel participants (n = 174).

The pattern for self-rated health also indicated a positive change from 68% rating their health as good in 1986 to 73% in 1996. Only 19 % of those with self-rated good health in 1986 had moved to bad health in 1996 while 52% of those rating their health as bad in 1986 rated their health as good ten years later (Figure 2). Preliminary results of the development in the reference area (not reported here) show a slightly higher starting level with 75% rating their health as good in 1986 ending up with 73% rating their health as good in 1999 and with similar movements between the groups. These figures correspond well with the national estimates indicating that approximately three quarters of the population rate their health as good, slightly lower for women than for men but no major changes during the study period [33].

Men's risk factor load developed more positively than women's, with 93 % maintaining low risk and 65% moving from high load to low compared to 83% and 47% among the women. For self-rated health we do not observe a significant difference between men and women. The lower educated had an initial higher risk load. There was no difference between higher and lower educated in keeping their risk factor load low (91 %, 89 %). However, the percentage decreasing their risk factor burden was higher among the lower educated, 62% compared to 48 %. The lower educated had an initial better health perception than the higher educated. Ten years later 86% of the higher educated had kept their self-rated good health from baseline compared to 76% among the lower educated. Still, it was the lower educated that improved their perceived health most with 57% of those rating their health as bad 1986 improving to good health in 1996 compared to 48% among the higher educated. The older age groups (50–60 in 1996) reduced their risk factor load considerably with 74% having a low risk load in 1996 compared to 54% in 1986, while the younger group remained at a low risk load level, 82% in 1996 compared to 83% in 1986. For the older group the pattern is similar for self-rated health with 74% rating their health as good in 1996 compared to 62% in 1986. For the younger age group self-rated good health reduced slightly from 76% to 71%.

#### Interaction between changes in risk factor load and self-rated health

In a multivariate logistic regression model we calculated the odds ratio for a combined positive outcome of self-rated good health and low risk factor load in 1996 based on self-rated health status and risk factor load in 1986, with good health as reference, adjusted for age, sex and educational level. Bad health at baseline reduces the chances of the positive outcome 10 years later by 70%. For those already perceiving their health as good and with a low risk factor load the odds of staying so are more than twice those starting with a high risk factor load despite their perceiving their health as good (p = 0.022). For men, when adding a high risk factor load to good health at baseline, their odds for perceiving both good health and having a low risk factor load ten years later (p = 0.909) do not decrease while for women adding high risk factor load to good health drastically reduces their odds of having both good health and low risk factor load ten years later (p = 0.007). Adding high risk load to bad health, on the other hand, seems to reduce the chances for men (p = 0.030) to have a positive outcome more than for women. Adding high risk to good health for the lower educated did not decrease their odds of having a positive outcome 10 years later as much as for the higher educated.

**Figure 3:**
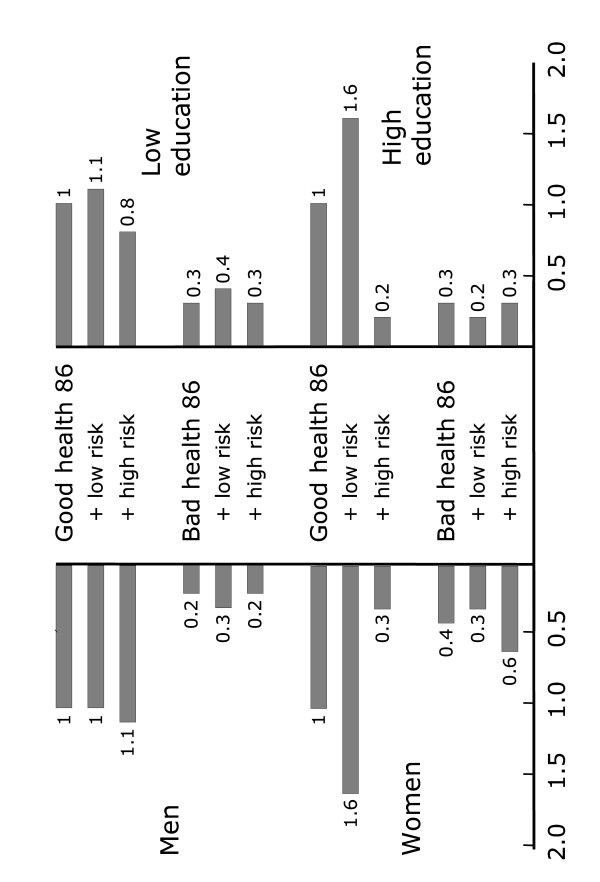
Odds ratio for self-rated good health and low risk factor load 1996 by sex, adjusted for, educational level and age group (left) and odds ratio for self-rated good health and low risk factor load 1996 by educational level, adjusted for gender and age group (right).

Figure 3 depicts by sex and education the prospects for a positive change into perceived good health and low risk through its odds ratio with reference to those with reported good health 1986 (OR = 1). Thus, for all four strata, when adjusting for age, sex/education reporting bad health already in 1986 has a strong negative impact on the chances of a positive change also for those with an initial low risk. For women and the highly educated the risk factor load seems especially important.

#### Methodological considerations

We acknowledge the small sample size as a limitation of our analysis of the panel participants, especially considering the risk of selection bias and differential misclassification. We observed that participants were somewhat older than non-participants, and that males were overrepresented among the young non-participants. However, there was no significant difference between participants and non-participants regarding biomedical risk factors or educational level. The small number of internally missing values for biomedical risk factors that was replaced with mean values is not likely to have influenced the results through differential misclassification. Internally missing values for other variables were classified with the aim to avoid overestimating the intervention effect.

Our decision to calculate the risk factor load based on the WHO guidelines for hypertension that were valid during the time of the intervention and not on the current guidelines can be debated. However, for us it makes sense to use the same levels of blood pressure that were used to inform people about their risk factors and formed the basis for the health counselling. Thus, for the participants it was these measures/levels that became the point of departure for their reflections about cardiovascular risk, their self-rated health as well as possible behavioural change. To us it is more of a hypothetical question if the potential for change had been greater or smaller if the new guidelines had been applied to our data.

We are also aware of the on-going scientific discussion about the additive or synergistic influence of the risk factors for cardio-vascular disease used in our study. This is reflected in for example the information given by the Swedish Medical Product Agency [34]. However, classifying high risk load as 2 or more risk factors has support from the American Heart Association [35], whose recommendation for absolute risk estimation is based on being ≥ 40 years or having ≥ 2 of the risk factors included in our risk load.

### The social context of the intervention programme

**Figure 4:**
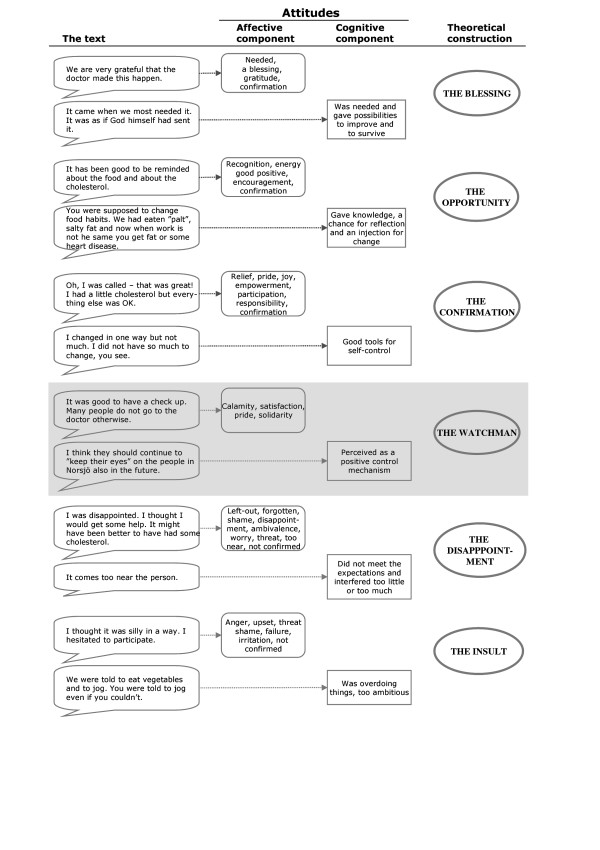
From attitudes and feelings to ideal types.

The Norsjö intervention programme took place in a specific social context and the outcome depended to a certain extent on this. The norms embedded in the programme had to face the existing norm systems in the Norsjö community, prescribing things about behaviour and attitudes as well as feelings. Our qualitative research interviews are the main data source for describing the norm systems and the attitudes and feelings regarding health and illness and how they have changed over time. This qualitative analysis was complemented with a quantitative analysis of 3 sets of questions from the 1996 survey questionnaire also focussing on health related attitudes and feelings. The generated "ideal types" presented in Figure 4 as a Grounded theory model of attitudes and feelings towards the intervention programme, are based solely on the qualitative interviews. Finally, we let the joint qualitative analysis help us to interpret and understand the interplay between risk factor load and perceived health.

#### Norm systems related to health and illness

##### Qualitative research interviews

When the intervention programme was launched in 1985 it challenged the view that health is a gift given by God/faith or formed by structural circumstances that ordinary people have limited possibilities to influence. The norms prescribed were such as: "You should be independent and manage on your own!" "You should go to work, even if....", "You should not allow yourself to feel ill" and "It's shameful to be ill". Our informants described a strong connection between health and work. Traditionally, work was seen as the goal and health more as a prerequisite for it – a means to an end. One informant recollected, "When I grew up, you were not supposed to waste any time. As soon as you sat down, you should make yourself useful and work with something". Health was regarded as a duty and ill-health as something to be denied until you could not do your work. A real man or woman was not supposed to give up even when tired, afraid, or ill. Consulting the doctor was traditionally seen as a last resort; "My dad used to say 'I won't go to the doctor until my head is under my arm'". Even if our informants described a shift towards allowing people today to care about their health, the old values were, to some extent, guiding current health seeking behaviour, especially for older people. The internalised norms are described as "It is inside our heads, even if we know it is wrong, this is what our parents told us". However, health has gradually become an end in itself, and work an important means of maintaining or improving it. "The young ones don't care so much about what other people think. They say that nobody will thank you if you are ill and still try to work."

#### Attitudes and feelings towards health and illness

##### Qualitative research interviews

The traditional strong connection between health and work influenced both attitudes and feelings. One informant described her mother saying; "She never complained, even if she was in pain. She struggled for a long time and was extremely enduring and I am proud of that". This *pride *in being strong was still there today; "Of course I have had the pain in my shoulder. It has been extremely painful but still it is nothing that has made me unable to work". Not being able to work created feelings of *shame*, especially among those above 50 years of age. We also observed a shift in that the psychological aspects of health had come to the fore. This development was perceived as both good and bad, "Today you are more allowed to 'feel ill'" but the disadvantage is that you "may not make enough effort and give up too easily". Our informants made a clear distinction between disease and the connected feelings. To be unhealthy was to be both physiologically and psychologically ill, i.e. when "something is wrong with the whole body". They would "rather have a disease and feel well" than "having a disease and feel bad" about it. A causal link between feelings and disease was also indicated: "There was something psychologically wrong that caused pain everywhere".

##### Survey questionnaire, 1996

When the group of panel participants were asked to rank some pre-determined aspects of health, a diversity of health perceptions were observed (Table 2). "Being physically fit and having energy" was equally important to "not being sick or having a disease" while "leading a healthy lifestyle" was least important. The results also indicated a more psychological view of health where "being psychologically fit" was regarded important, especially for the higher educated middle-aged men.

In a single survey question the panel participants were also asked if they thought that being long-term ill was shameful. A higher percentage of men (21%) than of women (15%) thought so, and it was more common for the lower (23%) than for the higher educated (14%) to think that it was shameful to be ill. No such difference was seen between age groups.

In an attempt to distinguish between feelings and disease we asked the panel participants to locate themselves in a two-by-two table indicating if they had a disease or not and if they felt good or bad. For the higher educated, 14% indicated that they had no disease but felt bad, compared to only 5% among the lower educated. On the other hand, among the lower educated 16% said they had a disease but still felt good, compared to only 8% among the higher educated. This could also partly be seen as a generation effect, with more educated youth, since a higher proportion of the older group said they had a disease but felt good while the younger group more often said they had no disease but still felt bad.

The research interviews suggested a transition regarding health related norm systems in the community. Our informants' reflections indicated that the former views on health being strongly related to work capability have been challenged and partly replaced by a health concept more related to feelings and a goal in itself. However, our survey questions revealed that the traditional norm systems may have had a greater impact on some groups (older and lower educated) but lost some of its influence on others.

#### Attitudes and feelings towards the intervention programme

When the intervention programme was introduced in 1985 it was mainly designed to influence social norms and attitudes related to certain cardiovascular risk behaviours and thereby reduce the total risk factor load for all inhabitants in the community. The programme was expected to decrease the load of certain risk factors and thus the risk for cardiovascular disease. The Norsjö model implied addressing and counselling everybody on an individual level at certain ages, while at the same time actively engaging the community in spreading messages about lifestyle changes, eating habits, physical activity and psychosocial conditions. The overall ambition was clear; "Better small changes in everyone, than large changes in a few". Even if there were considerable efforts to increase the community participation the main emphasis remained on life style changes as defined by the health care system [23].

##### Qualitative research interviews

Our informants were asked to reflect on both how they and others viewed the intervention programme in its early days, their own experience of the health examination and their views about the programme today. Their stories confirmed previous descriptions of a programme that was well established, accepted and able to start a dialogue with the population. A common problem of cardiovascular disease had been identified and made understandable to people, if not from the very start, at least during the process. The central role of the health examination screening and counselling was also confirmed. However, when specifically focussing on attitudes towards the programme, a pattern developed where we could identify both affective and cognitive components important for a discussion about why not everybody perceived the programme positively and how this may have influenced their disposition for action/behavioural change.

Figure 4 presents the range of identified attitude sets towards the programme exemplified with quotes from the interviews. To illustrate also our methodological approach the open codes for the affective components, expressed as feelings or emotions, are included. The cognitive aspects are summarised as hypotheses of how the affective components are related to people's reflections and evaluation of the programme. Finally we present a typology including six "ideal types. Together with information about the health related norm system these ideal types were used in the interpretation of the changes in risk factor load and self-rated health.

*"The Blessing" *is a metaphorical expression implying that people representing this attitude set saw the intervention as something bigger, outside themselves that came to their rescue. They knew something was wrong but did not know how to cope with it. They had not started to communicate their worries and not consulted the primary health care. They were influenced by the prescribed norms of not being allowed to "feel" and be concerned with your own health. When "the Blessing" appeared, they saw the implementers as necessary for improving their situation, and even for them to survive. They felt grateful and cognitively they also acquired knowledge to modify their risk behaviours. When successful, they felt grateful for the support.

*"The Opportunity" *followed the same line of thinking even if the feeling of relief was not so connected to the doctor or other external forces. This ideal type was more associated with pride and people's own choices and behaviour. The representatives of this ideal type were not hiding perceived illness. They tried to do something and made attempts to mobilise their own resources. The intervention programme gave them an opportunity to reflect and make constructive changes. When they succeeded they felt good and proud of themselves.

The third positive ideal type, "*The Confirmation"*, was influenced by both external and internal forces. The representatives regarded themselves as parts of a whole, in need of being confirmed. The feelings expressed were related to participation and empowerment. The intervention programme substantially increased their ability of self-control. The distinction between illness, sickness and disease was small for them. They did not necessarily feel a need for changing the targeted risk factors but felt recognised for being on the right track already.

The fourth ideal type, *"the Watchman"*, was still positive in nature. It represented an attitude set for people that viewed the programme as a common good to be proud of. It represented a general concern for the community and created a feeling of trust. The programme was compared to a regular check-up for cars.

The two negative "ideal types" included some degree of criticism towards the programme. The participants representing *"The Disappointment" *felt ignored and left out, in need of more help than the programme could offer. These persons may not have fitted into the risk groups identified by the programme but had other problems to attend to. They became disappointed because they had high expectations which were not met by the programme.

Even more vulnerable were those participants viewing the programme as *"the Insult"*. They expressed ambivalence towards the programme even if they may have applauded it at the start. Their participation was more based on feelings than on their own health problems. However, they may have had the targeted risk factors but felt that they could not meet the demands from the programme. They felt criticised and worried over not being able to do something about it. In this group there was also a greater suspicion about the collective ambition of the program.

A joint feature for the positive as well as the negative ideal types are the feeling of being seen and confirmed or not. The statement "it might have been better to have had some cholesterol" taken from the ideal type "The Disappointment" was interpreted as a strong indication of this.

#### Trustworthiness of the qualitative analysis

The qualitative analysis was mainly based on in-depth information from a purposive sample of individuals that represented the phenomena under study. In an ideal situation the decision about when to stop data collection is guided by level of saturation of the categories, hypotheses or theories under development [27]. In this study informants were sampled on the basis of being community members with different experiences of the intervention. In retrospect, one could of course wish that some more interviews had been performed, especially with men. Even if we during data collection felt that we had captured the variation in health related norms and attitudes and perceptions about the intervention, additional interviews might have deepened our understanding of gender aspects. However, one method of judging the trustworthiness of a qualitative analysis is through *member checking*. In this study, as part of the analytical process, preliminary results were presented and discussed with the health care personnel working with the intervention and with a scientific advisory group. This type of member checking sessions, together with the overall long-term research engagement, triangulation in terms of investigators, data collection methods, analytical approaches and theories, helped to strengthen our final interpretation.

#### Interpretation of the changes in risk factor load and self-rated health

The quantitative analysis showed that good health and low risk factor load at baseline were the best predictors of a positive outcome 10 years later and that bad health at baseline reduced the odds of an overall positive outcome by 70%. Our ideal types may not help us very much in explaining the stability over time on an individual level but "The Watchman" represents important attitude features on group/community level for maintaining both positive risk factor load and perceived good health.

However, the quantitative results also implied a larger polarisation process for men than women, where the distance between winners and losers was bigger for men. Men who felt good at baseline and had a high risk factor load had higher odds than women of a positive combined outcome 10 years later. A hypothesis generated from our Grounded Theory analysis is that males were overrepresented in the positive ideal type *"the Confirmation" *as well as in the negative type *"The Insult"*. The programme was probably also supported by their spouses helping them in their attempts to change life style and risk behaviour. Initially, mainly "male diseases" were targeted where men had more to gain. They may also have had more "embodied experience" of cardiovascular problems, motivating them for change [36]. Men may therefore have felt more relieved, empowered and proud when succeeding to change as well as more ashamed or more insulted when failing. Women do not seem to have been confirmed to the same extent and may therefore have been overrepresented in *"the Disappointment"*, wishing to have been seen even if not having any of the targeted risk factors. The feelings of disappointment would act as a barrier of moving towards the positive pole of self-rated health and even increased the risk of moving from low to high risk factor load.

The differences between educational groups may be looked upon in the same way. The intervention targeted changes in life style habits (fatty food, smoking and physical inactivity) that were more prevalent among the lower educated. This group was also more influenced by norms prescribing a strong association between work and health, creating barriers towards consulting the health care system before they were very ill. However, because of the intervention programme they were now invited to a check up, recognised and given some important tools for change if they were identified as having any of the included risk factors. They would then be overrepresented in all the positive ideal types if they felt good at baseline but in the negative types if they perceived their health as bad.

## Conclusion

The Norsjö intervention programme brought about changes in everyday life in the community. The programme influenced existing health related norm systems and more directly attitudes and behaviours. Cognitive influences were often preceded by affective feelings. Most people have maintained a low risk factor load and a positive perception of health during the 10 year period. From a prevention point of view that in itself is a good outcome. However, our analysis also indicated that more people have improved than deteriorated their situation regarding both perceived health and risk factor load during the period which could be regarded as an added benefit of the programme. The "ideal types" helped us understand possible mechanisms behind the observed polarisation for men and lower educational groups and the limitations of the influence of the intervention programme.

The multi-disciplinary evaluation of the Norsjö intervention programme set out to include effect as well as process components [37]. So far the evaluations have mainly focussed on the manifest (intended) functions of the programme resulting in an overall positive assessment of the bio-medical outcome as well as the equity and community participation aspects. In this study we used the manifest function of a risk factor reduction to analyse also latent functions related to self-rated or perceived health. Latent functions are those that are neither intended nor immediately recognised by the actors involved. They can be both functional (positive) and dysfunctional (negative). Thus, from our joint analysis, we are prepared to conclude that the worry and anxiety that the intervention efforts may have generated, measured through level of self-rated bad health, have not taken precedence over the manifest and functional consequences. By including a qualitative approach focussing on the participants' attitudes towards the programme, we have tried to contribute to the dissection of "the black box" of community interventions [38]. We have illustrated the complexity in the interaction between feelings and willingness/possibility to adopt new health related norm systems for preventing future disease. Our observation of a socially and gender differentiated intervention effect suggests a need to test new intervention strategies. Future community interventions may benefit from targeting more directly those who in combination with high risk factor load perceive their health as bad and to make all participants feel seen, confirmed and involved.

## Competing interests

The author(s) declare that they have no competing interests.

## Authors' contributions

ME developed and carried out the follow-up questionnaire as well as the qualitative interviews. She was responsible for the design of the study and for drafting the manuscript. The statistical analysis was performed in collaboration with HS, LW and SW, while the qualitative analysis was made in collaboration with LD. HS also contributed with critical revisions of the methodological and results sections. LW and LD contributed in the discussion of the design, participated in part of the data collection, were involved in the quantitative and qualitative analysis and have made important contributions to the article. SW participated substantially in the analysis and interpretation of the survey data and contributed with critical revisions of the article. All authors have read and approved the final manuscript.

## Pre-publication history

The pre-publication history for this paper can be accessed here:


